# Reducing Smad3/ATF4 was essential for Sirt1 inhibiting ER stress-induced apoptosis in mice brown adipose tissue

**DOI:** 10.18632/oncotarget.14035

**Published:** 2016-12-20

**Authors:** Zhenjiang Liu, Huihui Gu, Lu Gan, Yatao Xu, Fei Feng, Muhammad Saeed, Chao Sun

**Affiliations:** ^1^ College of Animal Science and Technology, Northwest A&F University, Yangling, Shaanxi, 712100, China

**Keywords:** Sirt, ATF, Smad, apoptosis, brown adipocytes

## Abstract

Sirtuin 1 (Sirt1) promotes adaptive thermogenesis by controlling the acetylation status of enzymes and transcriptional factors in interscapular brown adipose tissue (iBAT). However, the effects of Sirt1 on endoplasmic reticulum (ER) stress and apoptosis of iBAT remain elusive. In this study, the mRNA levels of *Sirt1* and thermogenesis genes were reduced but the genes related with ER stress were elevated in iBAT of high-fat diet (HFD)-induced obese mice. Moreover, ER stress further inhibited mRNA level of *Sirt1* and triggered brown adipocyte apoptosis *in vitro* and *in vivo*. Further analysis revealed that *Sirt1* overexpression alleviated ER stress-induced brown adipocyte apoptosis by inhibiting Smad3 and ATF4. In addition, Smad3 bound to *ATF4* promoter region and positively transcriptional regulation of *ATF4*. Our data also confirmed that Sirt1 reduced early apoptotic cells and blocked the mitochondrial apoptosis pathway by directly interacting with ATF4. Furthermore, Sirt1 attenuated tunicamycin-induced cold intolerance and elevating thermogenesis by inhibiting ER stress and apoptosis in iBAT. In summary, our data collectively revealed Sirt1 reduced ER stress and apoptosis of brown adipocyte *in vivo* and *in vitro* by inhibiting Smad3/ATF4 signal. These data reveal a novel mechanism that links Sirt1 to brown adipocyte apoptosis.

## INTRODUCTION

Sirtuin Type 1 (Sirt1), a member of sirtuins family, is well known for its deacetylation regulation of cell cycle, energy homeostasis and apoptosis in adipose tissue [1–3]. Sirt1 adipocyte-specific knockout mice present low-grade chronic inflammation along with glucose intolerance and insulin resistance [4]. Our previous study confirms that Sirt1 decreases white adipose inflammation (WAT) via interacting with Akt2 and activating mTOR/S6K pathway [5]. Sirt1 also reduces fat accumulation and improves whole-body energy expenditure in white adipocytes [2, 6]. Interscapular brown adipose tissue (iBAT) is a key organ in the regulation of energy expenditure and thermogenesis [7, 8]. Recent studies show that Sirt1 increases metabolic activity and promotes the brown remodeling of white adipocytes [9–11]. Moreover, Sirt1 deficient exacerbates lower thermogenic activity and aggravates mitochondrial dysfunction in brown adipose tissue of diet-induced obese mice [12]. Further analyses showed a decrease in mitochondrial content associated with lower UCP1 level in the iBAT of Sirt1 deficient mice [12]. This is in line with the strong influence of Sirt1 on PPARα target genes, including *UCP1* in iBAT [13, 14]. However, the effects of Sirt1 on brown adipocyte apoptosis have not been established.

Endoplasmic reticulum (ER) stress is caused by dysfunction of ER homeostasis and exacerbates various diseases including diabetes, fatty liver, and chronic obstructive pulmonary disease [15]. Recent study shows that overexpression of *Sirt1* attenuates hepatic steatosis and ER stress condition, ameliorates insulin resistance, and restores glucose homeostasis [16]. The overload calorie intake causes hypothalamic ER stress and reduces iBAT thermogenesis [17]. In addition, ER stress also triggers insulin resistance and damages adaptive thermogenesis via the down-regulation of mitochondrial mtDNA in iBAT [18]. Apoptosis, or programmed cell death, is essential for maintaining cellular homeostasis. Studies show that ER stress is correlated with apoptosis in adipocytes [19, 20]. However, the molecular mechanisms of Sirt1 on ER stress-induced apoptosis in brown adipocytes are still unclear.

In this study, we demonstrated that Sirt1 reduced ER stress-induced apoptosis in brown adipose tissue. Our data showed that Sirt1 functioned through Smad3/ATF4 signal and also physically interacted with ATF4. These results imply that Sirt1 could be used as a new therapeutic means to prevent and treat obesity and type 2 diabetes.

## RESULTS

### Sirt1 was reduced along with increased ER stress in brown adipose tissue of obese mice

**Figure 1:**
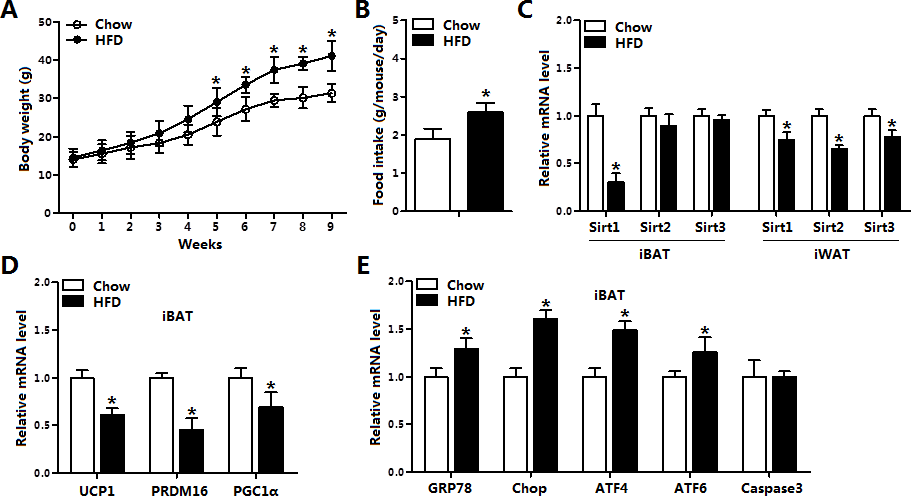
Sirt1 was reduced along with increased ER stress in brown adipose tissue of obese mice (**A**) Body weight of mice fed on high fat diet (HFD) and chow diet (*n* = 6); (**B**) Food intake of mice fed on HFD and chow diet (*n* = 6); (**C**) The mRNA expression of *Sirt1*, *Sirt2*, *Sirt3* in interscapular brown adipose tissue (iBAT) and inguinal white adipose tissue (iWAT) of mice fed on HFD or chow diet (*n* = 6); (**D**) The mRNA expression of *UCP1*, *PRDM16* and *Cidea* in iBAT of mice fed on HFD or chow diet (*n* = 6); (**E**) The mRNA expression of *GRP78*, *Chop*, *ATF4*, *ATF6* and *Caspase3* in iBAT of mice fed on HFD or chow diet (*n* = 6). Values are means ± SEM, *p < 0.05 compared with the control group.

As shown in Figure 1A, body weight was increased after HFD for 9 weeks along with the elevation of food intake (Figure 1A and 1B). HFD treatment reduced the mRNA level of *Sirt1* in both inguinal white adipose tissue (iWAT) and interscapular brown adipose tissue (iBAT) (Figure 1C), but did not change the expression of *Sirt2* and *Sirt3* in iBAT compared with those in iWAT (Figure 1C). We then measured the mRNA levels of *UCP1*, *PRDM16* and *PGC1-α* in iBAT, data showed HFD significantly inhibited the mRNA levels of these thermogenesis marker genes (Figure 1D). Additionally, our result demonstrated HFD triggered ER stress by increasing the expression of *GRP78*, *Chop*, *ATF4* and *ATF6* but not *Caspase3* (Figure 1E). Thus, HFD impaired the function of brown adipose tissue and activated ER stress accompanied with the reduction of *Sirt1*.

### ER stress decreased Sirt1 and increased apoptosis in the brown adipose tissue

**Figure 2:**
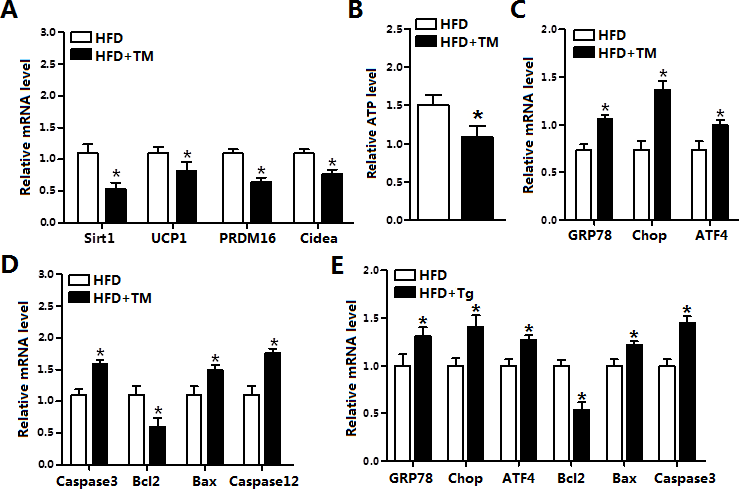
ER stress decreased Sirt1 and increased apoptosis in the brown adipose tissue (**A**) Relative mRNA levels of *Sirt1*, *UCP1*, *PRDM16* and *Cidea* in iBAT of mice fed on HFD injected with or without tunicamycin (TM, 1 μg/g) (*n* = 6); (**B**) ATP level in iBAT of mice fed on HFD injected with or without TM (*n* = 6); (**C**) Relative mRNA levels of *GRP78*, *Chop* and *ATF4* in iBAT of mice fed on HFD injected with or without TM (*n* = 6); (**D**) Relative mRNA levels of *Caspase3*, *Bcl2*, *Bax* and *Caspase12* in iBAT of mice fed on HFD injected with or without TM (*n* = 6); (**E**) Relative mRNA levels of *GRP78*, *Chop*, *ATF4*, *Bcl2*, *Bax* and *Caspase3* in iBAT of mice fed on HFD injected with or without Tg (*n* = 6). Values are means ± SEM, *p < 0.05 compared with the control group.

To investigate role of ER stress in diet-induced obese mice, we used tunicamycin (TM) to inject HFD male mice. Result indicated TM injection markedly reduced the mRNA levels of *Sirt1*, *PRDM16*, *Cidea* and *UCP1* (Figure 2A). And the ATP concentration of iBAT was decreased after TM treatment (Figure 2B). As expected, ER stress markers *GRP78*, *Chop* and *ATF4* were all elevated after TM injection (Figure 2C). Further analysis demonstrated TM injection triggered apoptosis of iBAT by increasing the levels of *Caspase3*, *Bax* and *Caspase12*, which suggested that TM-induced ER stress not only damaged iBAT thermogenesis but also induced brown adipocyte apoptosis (Figure 2D). Additionally, thapsigargin (Tg)-induced ER stress further confirmed that ER stress induced apoptosis in iBAT (Figure 2E). These data collectively revealed brown adipose apoptosis have a connection with ER stress.

### ER stress inhibited Sirt1 expression and damaged UCP1 function in brown adipocytes

**Figure 3:**
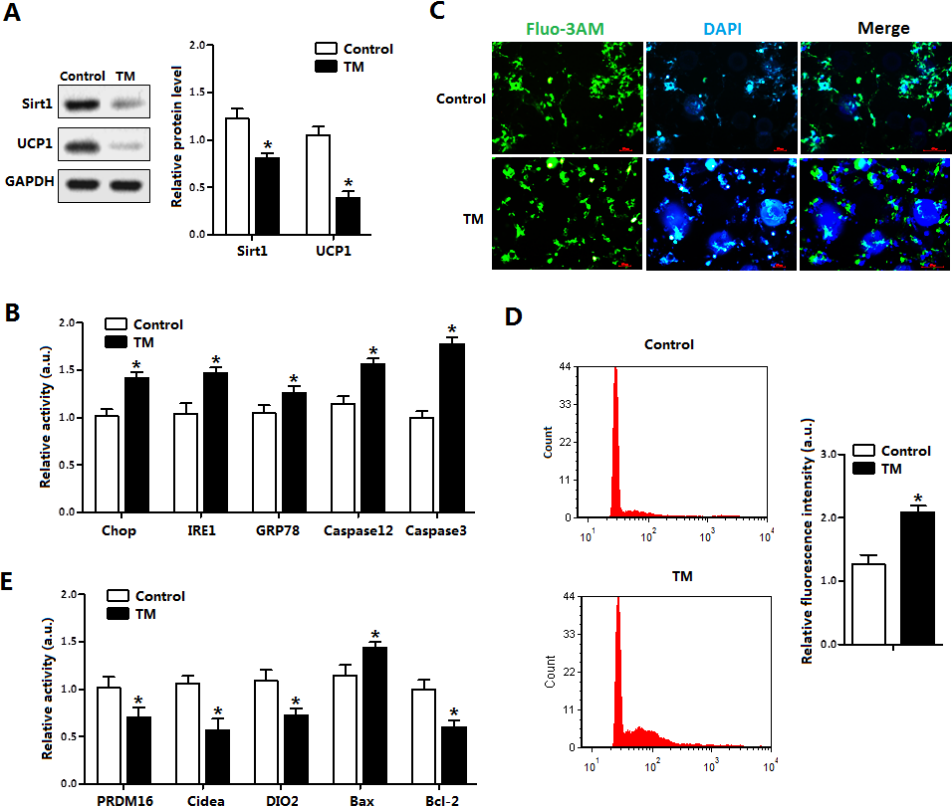
ER stress inhibited Sirt1 expression and damaged UCP1 function in brown adipocytes (**A**) Protein level of Sirt1 and UCP1 with tunicamycin (TM, 1 μg/mL) incubation for 12 h in brown adipocytes (*n* = 3); (**B**) Protein levels of Chop, IRE1, GRP78, Caspase12 and Caspase3 of brown adipocytes with TM incubation for 12 h (*n* = 3); (**C**) Cytosolic Ca^2+^ in brown adipocytes with TM incubation for 12 h (*n* = 3); (**D**) Flow cytometry (FCM) analysis of Cytosolic Ca^2+^ in Figure 3C (*n* = 3); (**E**) Protein level of PRDM16, Cidea, DIO2, Bax and Bcl-2 of brown adipocytes incubated with TM for 12h. Protein level was measured by ELISA test (*n* = 3). Values are means ± SEM, *p < 0.05 compared with the control group.

The effects of ER-stress on apoptosis in brown adipocytes are shown in Figure 3. The TM treatment for 12 h decreased the levels of Sirt1 and UCP1 (Figure 3A). Consistent with *in vivo* study, TM-induced ER stress triggered brown adipocytes apoptosis by elevating the protein levels of Chop, Caspase 3 and Caspase 12 (Figure 3B). Elevation of cytosolic Ca^2+^, an indicator of ER stress induced apoptosis, was observed after TM treatment (Figure 3C and 3D). ELISA detection of apoptosis proteins verified that TM triggered brown adipocytes ER stress (Figure 3E).

**Figure 4:**
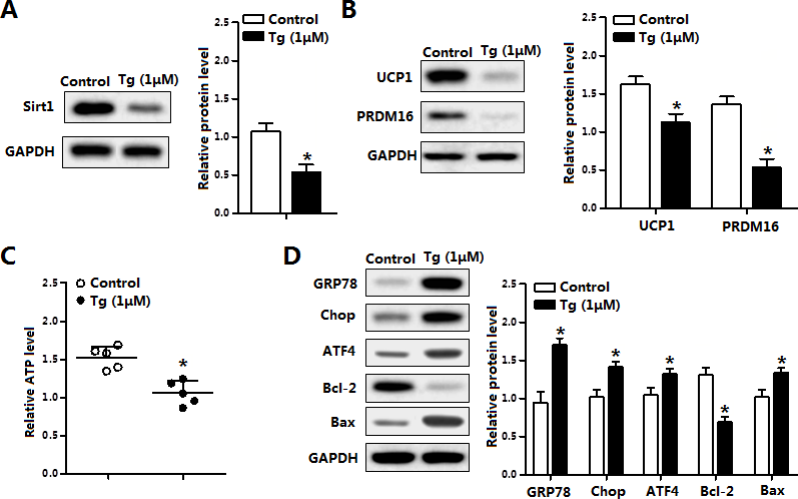
Thapsigargin (Tg) induced ER stress triggered brown adipocytes apoptosis and reduced UCP1 (**A**) Protein of Sirt1 of brown adipocytes treated with thapsigargin (Tg, 1 μM) for 12 h (*n* = 3); (**B**) Protein level of UCP1 and PRDM16 of brown adipocytes treated with 1 μM Tg for 12 h (*n* = 3); (**C**) ATP level of brown adipocytes treated with 1 μM Tg for 12 h (*n* = 3); (**D**) Protein level of GRP78, Chop, ATF4, Bcl-2 and Bax of brown adipocytes treated with 1 μM Tg for 12 h (*n* = 3). Values are means ± SEM, *p < 0.05 compared with the control group.

Further Tg-induced ER stress also triggered brown adipocytes apoptosis. As shown in Figure 4A, Tg treatment reduced the protein levels of Sirt1, UCP1 and PRDM16 (Figure 4B). Cellular ATP level was decreased as it is in the *in vivo* experiment (Figure 4C). Brown adipocytes apoptosis was also along with the elevation of ER stress markers (Figure 4D). The above data demonstrated ER stress blocked the thermogenesis and triggered apoptosis of brown adipocytes.

### Sirt1 decreased brown adipocytes apoptosis and reduced ATF4 level

**Figure 5:**
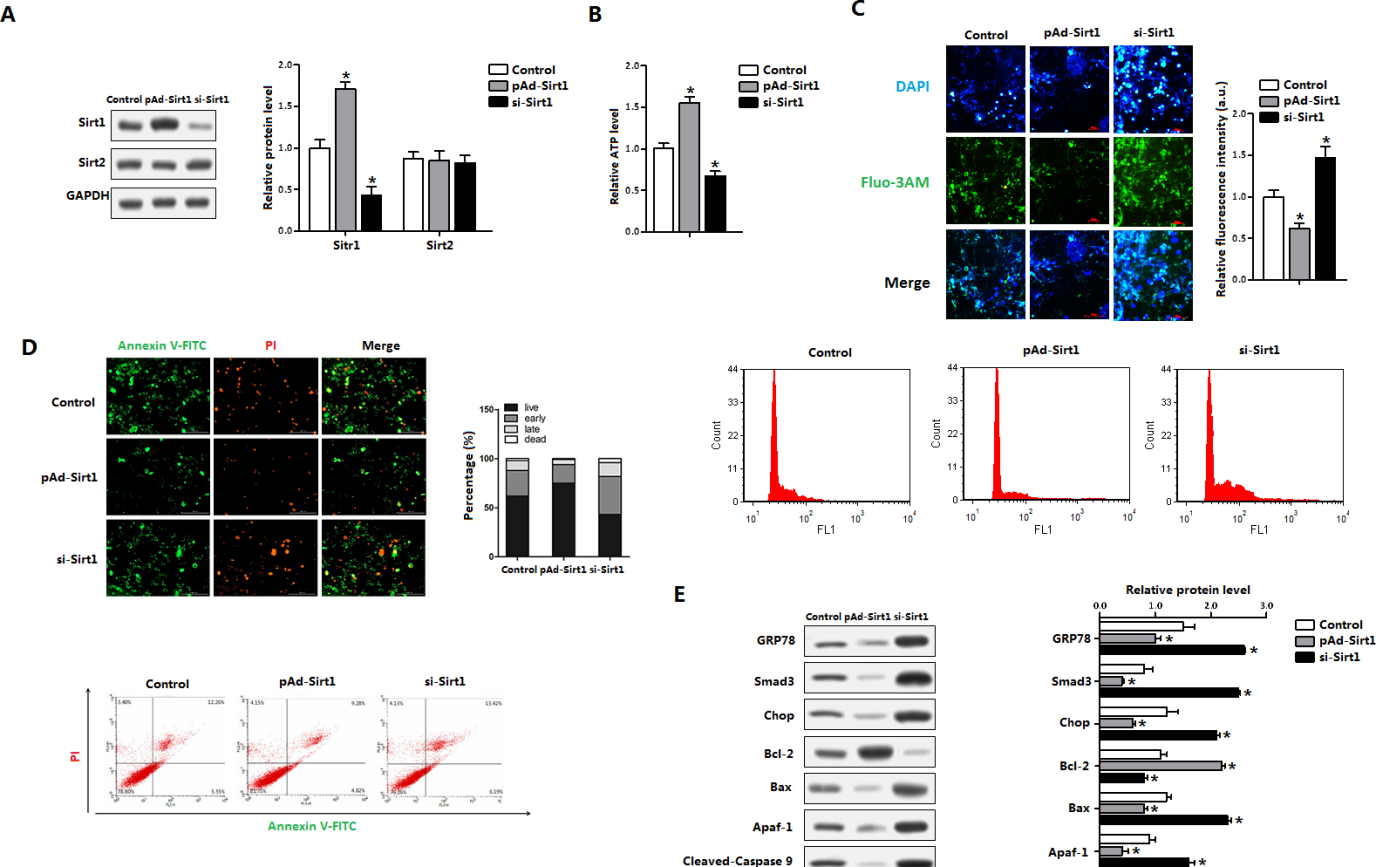
Sirt1 decreased brown adipocytes apoptosis and reduced ATF4 level Brown adipocytes were pre-infected with recombinant vectors of Sirt1 (pAd-Sirt1 or si-Sirt1) for 48 h. *n* = 3 for each treatment. (**A**) Protein level of Sirt1 and Sirt2 in brown adipocytes; (**B**) ATP level of brown adipocytes; (**C**) Cytosolic Ca^2+^ concentration and Flow cytometry (FCM) analysis of Cytosolic Ca^2+^ in brown adipocytes; (**D**) Annexin V-FITC/PI double staining and flow cytometry analysis of brown adipocyte apoptosis stages; (**E**) Protein levels of GRP78, Smad3, Chop, Bcl-2, Bax, Apaf-1, Cleaved-Caspase9 and Cleaved-Caspasw3 in brown adipocytes. pAd-Sirt1: recombinant adenovirus over-expression vector of Sirt1, si-Sirt1: recombinant lentiviral interference vector of Sirt1. Values are means ± SEM. *p < 0.05 compared with the control group.

We next addressed whether Sirt1 ameliorated ER stress and apoptosis in mice brown adipocytes. As shown in Figure 5A, cells were infected with the recombinant adenovirus vector of Sirt1 (pAd-Sirt1) led to an increase of Sirt1, but not Sirt2 (Figure 5A); and elevated the cellular ATP level (Figure 5B). pAd-Sirt1 decreased the intracellular Ca^2+^ level which was elevated under ER stress condition (Figure 5C), suggesting that brown adipocytes apoptosis was reduced. Further Annexin V staining measurement indicated that *Sirt1* decreased the apoptotic cells and increased the number of live cells (Figure 5D). Those changes were correlated with the protein reductions of GRP78, Chop and ATF4 (Figure 5E). And the mitochondrial apoptosis marker proteins Bax, Apaf-1 and cleaved-caspase3 were also decreased (Figure 5E). These data collectively suggested that Sirt1 was associated with ER stress and apoptosis of brown adipocytes.

### Smad3 promoted ATF4 transcription in brown adipocytes

**Figure 6:**
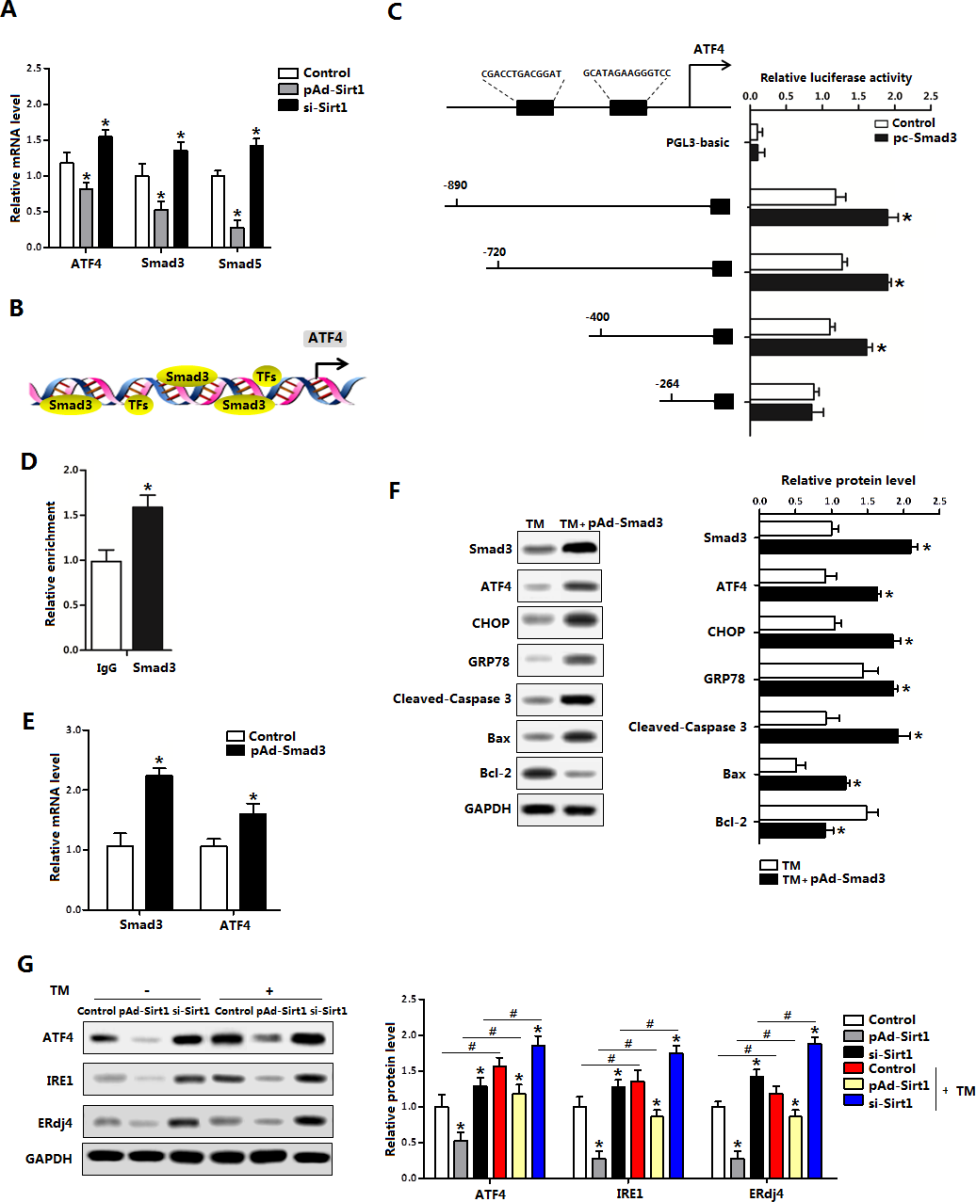
Smad3 promoted ATF4 transcription in brown adipocytes (**A**) Relative mRNA levels of *ATF4*, *Smad3* and *Smad5* of brown adipocytes pre-infected with pAd-Sirt1 or si-Sirt1 for 24 h (*n* = 3); (**B**) Predicate translation factors of ATF4 by using Genomatix software; (**C**) Dual luciferase reporter assay of ATF4 and Smad3. HEK293 cells were transfected with PGL3-basic or PGL3-ATF4 plasmids, and pc-Smad3 plasmid (*n* = 3); (**D**) ChIP analysis between ATF4 and Smad3 (*n* = 3); (**E**) Relative mRNA levels of *Smad3* and *ATF4* with pAd-Smad3 infection of brown adipocytes (*n* = 3); (**F**) Protein levels of Smad3, ATF4, CHOP, GRP78, Cleaved-Caspase3, Bax and Bcl-2 of brown adipocytes pre-incubated with TM and treated with pAd-Smad3 or not (*n* = 3); (**G**) Protein levels of ATF4, Smad3, IRE1 and ERdj4 of brown adipocytes pre-incubated with TM, and infected with pAd-Sirt1 or si-Sirt1 (*n* = 3). pAd-Smad3: recombinant adenovirus over-expression vector of Smad3, pc-Smad3: Overexpression plasmid of Smad3; pAd-Sirt1: recombinant adenovirus over-expression vector of Sirt1, si-Sirt1: recombinant lentiviral interference vector of Sirt1. Values are means ± SEM. *p < 0.05, ^#^*p* < 0.05 compared with the control group.

We next explored whether Sirt1 had transcription regulation on ER stress. Interestingly, our data showed pAd-Sirt1 treatment significantly reduced *ATF4*, *Smad3* and *Smad5* levels (Figure 6A). In addition to previous known role of Smad3 in the promotion of ER stress, two potential binding sites of Smad3 on *ATF4* promoter region were found with Genomatix software analysis (Figure 6B). And further measurements revealed the binding site, 264 bp-400 bp upstream of the initiation sites of *ATF4* functioned (Figure 6C). The transcriptional regulation between Smad3 and ATF4 was also verified by the ChIP measurement (Figure 6D). Figure 6E showed pAd-Smad3 significantly increased the mRNA levels of *Smad3* and *ATF4* in brown adipocytes (Figure 6E). In addition, CHOP and GRP78 protein levels were elevated after Smad3 treatment compared with control group in the brown adipocytes which had been pre-incubated with TM (Figure 6F). Our data also demonstrated that Smad3 aggravated brown adipocytes apoptosis (Figure 6F). Western blot analysis showed Sirt1 ameliorated TM-induced ER stress, involved in the reduction of ATF4 (Figure 6G). Together, these findings strongly suggested Smad3 controls ER stress by elevating *ATF4* transcription level.

### Sirt1 directly interacted with ATF4 in the regulation of brown adipocytes ER stress

**Figure 7:**
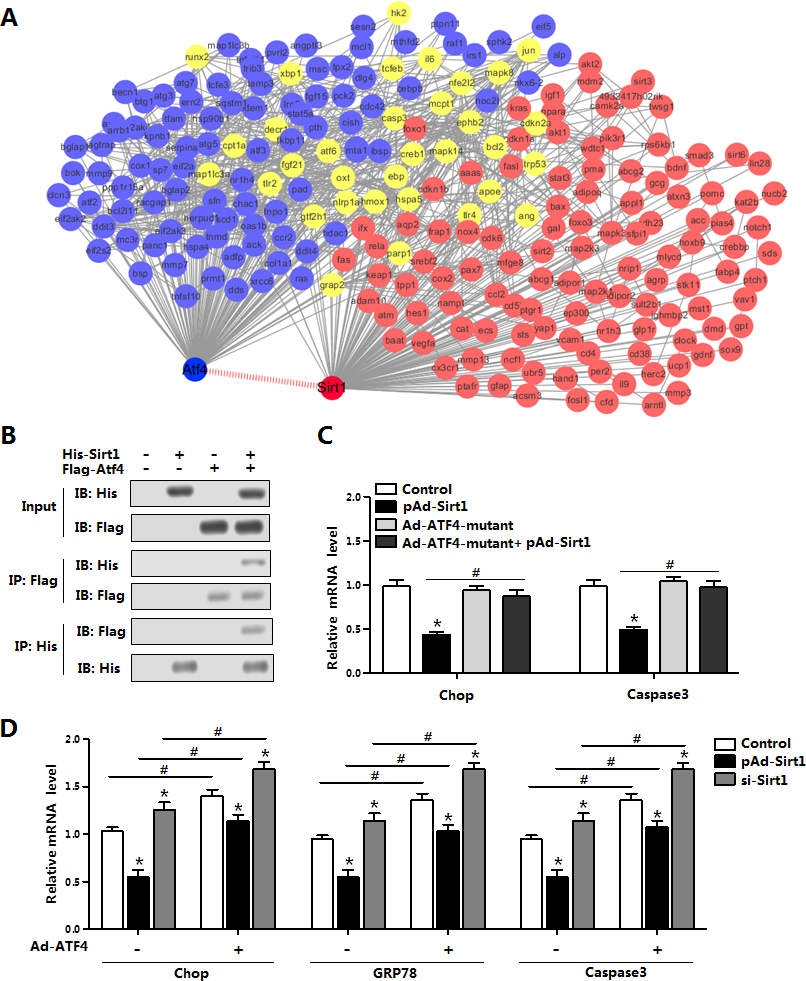
Sirt1 directly interacted with ATF4 in the regulation of brown adipocytes ER stress (**A**) Bioinformatics analysis between ATF4 and Sirt1 (*n* = 3); (**B**) Co-IP analysis between Sirt1 and ATF4. Cells were transfected with His-Sirt1 or Flag-Atf4 in HEK293T cells (*n* = 3); (**C**) Relative mRNA level of *Chop* and *Caspase3* in brown adipocytes infected with Ad-ATF4, pAd-Sirt1 or Ad-ATF4-mutant vectors for 24 h (*n* = 3); (**D**) Relative mRNA levels of *Chop*, *GRP78* and *Caspase3* in brown adipocytes pre-infected with pAd-Sirt1 or si-Sirt1, and combined infected with or without Ad-ATF4 (*n* = 3). His-Sirt1: Over-expression plasmid vector of pc-DNA3.1-His-Sirt1 (His-Sirt1), Flag-Atf4: Over-expression plasmid vector of pc-DNA3.1-Flag-Atf4 (Flag-Atf4), Ad-ATF4: recombinant adenovirus over-expression vector of ATF4, Ad-ATF4-mutant: recombinant adenovirus over-expression vector of mutant ATF4, pAd-Sirt1: recombinant adenovirus over-expression vector of Sirt1, si-Sirt1: recombinant lentiviral interference vector of Sirt1. Values are means ± SEM. *p < 0.05, ^#^*p* < 0.05 compared with the control group.

As the mRNA levels of *ATF4* and *Smad3* were all reduced with Sirt1 treatment (Figure 6A), we next examined whether Sirt1 reduced ER stress by the interaction with ATF4 on protein level. Firstly with the bioinformatics analysis and previous data sheet, we predicted Sirt1 could directly interact with ATF4 (Figure 7A). Then by protein-protein measurement, we found Sirt1 protein interacted strongly with ATF4 in transfected HEK293T cells (Figure 7B). Over-expression of Sirt1 significantly decreased the mRNA levels of *Chop* and *Caspase3* in the brown adipocytes (Figure 7C). The expression of *Chop* and *Caspase3* had no change in the cells treated with Ad-ATF4-mutant; and the co-treatment of pAd-Sirt1 and Ad-ATF4-mutant had no effects on the expression of *Chop* and *Caspase3* either (Figure 7C). In addition, co-treatment of pAd-Sirt1 and Ad-ATF4 attenuated the effects of Ad-ATF4 on ER stress and brown adipocytes apoptosis (Figure 7D). Thus these data suggested Sirt1 directly bind with ATF4, and ATF4 functioned in a Sirt1-dependent manner in the negative regulation of ER stress and apoptosis in brown adipocytes.

### Sirt1 alleviated ER stress-induced cold intolerance in mice

**Figure 8:**
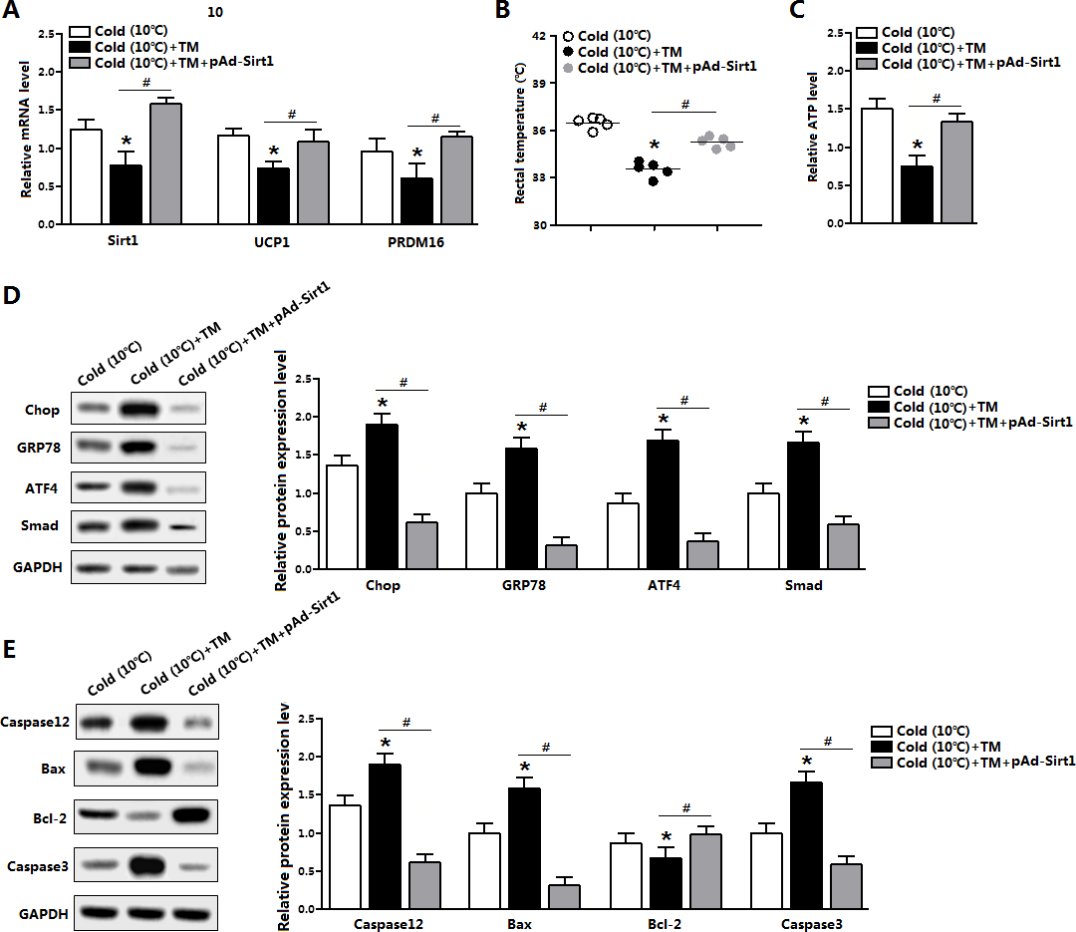
Sirt1 alleviated ER stress-induced cold intolerance in mice (**A**) Relative mRNA level of *Sirt1*, *UCP1* and *PRDM16* in the brown adipose tissue of mice kept under 10°C for 2 days, and injected with TM or not in the mice pre-treated with pAd-Sirt1 (*n* = 6); (**B**) Temperature of mice kept under 10°C for 2 days, and injected with TM or not in the mice pre-treated with pAd-Sirt1 (*n* = 6); (**C**) ATP level of mice kept under 10°C for 2 days, and injected with TM or not in the mice pre-treated with pAd-Sirt1 (*n* = 6); (**D**) Protein levels of Chop, GRP78, ATF4, and Smad3 of mice kept under 10°C for 2 days, and injected with TM or not in the mice pre-treated with pAd-Sirt1 (*n* = 6); (**E**) Protein levels of Caspase12, Bax, Bcl-2 and Caspase3 of mice kept under 10°C for 2 days, and injected with TM or not in the mice pre-treated with pAd-Sirt1 (*n* = 6). pAd-Sirt1: over-expression recombinant adenovirus vector of Sirt1. Values are means ± SEM. *p < 0.05, ^#^*p* < 0.05 compared with the control group.

To test the effect of Sirt1 on brown adipose function, we used a model of cold exposure mice. Cold exposure markedly elevated the mRNA level of *Sirt1*, and increased the levels of *UCP1* and *PRDM16* (data not shown). TM injection inhibited *Sirt1* expression and damaged brown adipose function indicated by the reducing of UCP1 and PRFM16 expression (Figure 8A). Extrinsic expression of Sirt1 restored the mRNA levels of *UCP1* and *PRDM16* (Figure 8A). Rectal temperature was decreased after TM injection, but Sirt1 treatment significantly recovered this decreasing temperature (Figure 8B). Consistently, brown adipose ATP level was also increased after Sirt1 injection (Figure 8C). Moreover, the Sirt1 treatment reduced the protein levels of GRP78, Chop, ATF4 and Smad3 (Figure 8D); these proteins were drastically increased after TM injection (Figure 8D). Consistently, Sirt1 reduced ER stress-induced apoptosis under cold expose condition (Figure 8E). Thus, Sirt1 promoted brown adipose function and attenuated ER stress in both diet-induced obese mice and cold-exposured mice.

## DISCUSSION

Brown adipose tissue plays important roles in adaptive thermogenesis (nonshivering thermogenesis) for the maintenance of basic body temperature and heat balance energy against cold [21, 22]. Stress-related alterations in endoplasmic reticulum, such as the unfolded protein response (UPR), are associated with obesity [23]. Studies show that UPR can activate IRE1α-XBP1 pathway and upregulate UCP1 in brown adipocytes [24]. Our previous studies also show that regulation of adipose tissue apoptosis has been a potential for treating obesity in decades [25–28]. In this study, we confirmed that TM induced ER stress and apoptosis in mice brown adipose tissue. Our results in this study showed that ER stress reduced *Sirt1* mRNA level in brown adipose tissue. These findings are consistent with published reports that demonstrate Sirt1 is closely related with ER stress in adipocytes [13, 29, 30].

The disturbance of intracellular Ca^2+^ signal has an important role that connection ER stress to mitochondrial functions [31]. A consequent mitochondrial Ca^2+^ overload causes alterations of the morphology of mitochondria and triggers cell apoptosis [32]. Here our data demonstrate that ER stress caused the disturbance of Ca^2+^ distribution and triggered apoptosis with increased *ATF4* mRNA level in brown adipocyte. Moreover, Sirt1 inhibited ATF4 and eliminated ER stress-induced apoptosis in brown adipocytes. Our results notably revealed the relationships between Sirt1, ER stress and mitochondrial apoptosis in brown adipose tissue, although the interaction between ER and mitochondria has been studied in brown adipose tissue [32, 33]. Moreover, Sirt1 is a crucial regulator of TGF-beta/Smad3 signal [34]. Studies show that up-regulation of Sirt1 deacetylase activity attenuates TGF beta-induced renal fibrosis and hepatocyte apoptosis through inhibiting Smad3 signal [35–37]. Here, we showed that Smad3 bind to the *ATF4* promoter region that promoted *ATF4* transcription, though few studies imply the relationship of Smad3 and ATF4 [38, 39].

Given the importance of brown adipose tissue for basal thermogenic energy expenditure, we investigated the molecular mechanisms of Sirt1 in the apoptotic responses of brown adipocytes. Thus, our results suggest that Sirt1 directly inhibits brown adipocyte apoptosis by regulating the interaction between ER and mitochondria, and furthermore this inhibitory effect is through reducing the Smad3/ATF4 signal. The role of ATF4, one of the upstream regulators of CHOP and a pro-apoptotic factor during cellular stress, is well documented [40]. It has been found that ATF4 mediates ER stress-induced cell death in tumor cells treated with the chemotherapeutic agents [41, 42]. Notably, we also found that Sirt1 inhibited brown adipocyte apoptosis by directly interaction with ATF4. Consequently, more molecular mechanisms of Sirt1 on brown adipocytes apoptosis warrant further studies.

**Figure 9:**
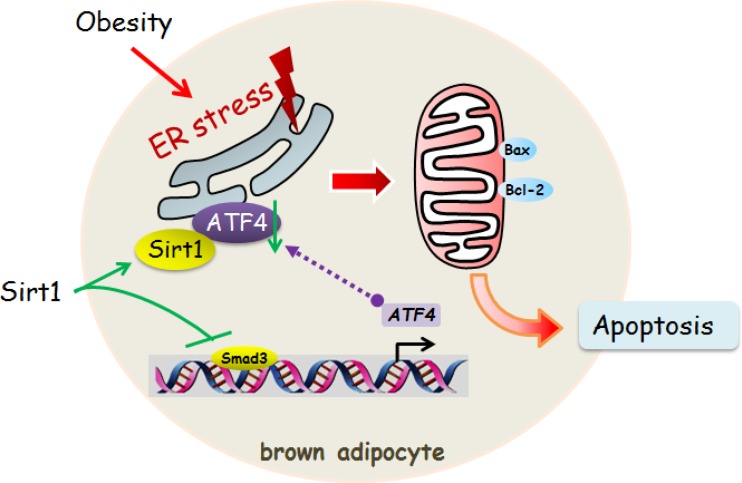
Sirt1 inhibited ER stress-induced brown adipocyte apoptosis through the Smad3/ATF4 signal Sirt1 inhibited ER stress-induced brown adipocyte apoptosis and directly binding with ATF4. Smad3 was a novel transcriptional activator of ATF4 in alleviating ER stress and apoptosis of brown adipocytes.

In summary, we found that Smad3 was a novel transcriptional activator of ATF4 in elevating ER stress and apoptosis of brown adipocytes. Moreover, our data provide compelling evidence that Sirt1 inhibited ER stress-induced brown adipocyte apoptosis through reducing Smad3/ATF4 signal (Figure 9). Our results contribute to further understand of the regulatory mechanisms of adipocyte apoptosis in the development of novel approaches to prevent and treat obesity.

## MATERIALS AND METHODS

### Animal study

Six-week-old C57BL/6J male mice were purchased from the Laboratory Animal Center of the Fourth Military Medical University (Xi’an, China). Mice handling protocols were conducted following the guidelines and regulations approved by the Animal Ethics Committee of Northwest A&F University (Yangling, China).

In diet induced obesity study, mice were placed on high fat diet (HFD, fat provides 60% of the total energy) for 9 weeks, control mice were fed with a standard chow diet (fat provides 10% of the total energy). Body weight and food intake of mice were recorded weekly. Mice were provided *ad libitum* water and the animal room was maintained at 25 ± 1°C, humidity at 55 ± 5%, and 12 h light/dark cycles. After 9 weeks of HFD feeding, the HFD + tunicamycin (TM, Sigma, St. Louis, MO, USA) group received intraperitoneal injection of 1 μg/g TM dissolved in dimethylsulphoxide (DMSO, Sigma, St. Louis, MO, USA) and diluted with PBS, or vehicle for 3 days; And HFD + thapsigargin (Tg, Sigma, St. Louis, MO, USA) group received intraperitoneal injection of 2 μM Tg for 3 days.

For cold exposure experiment, ten-week-old mice were exposed at 10°C for 2 days; then the recombinant adenovirus vector of Sirt1 (pAd-Sirt1) was intravenous injection for 7 days followed by the intraperitoneal injection of 1 μg/g TM for 3 days. Mice were then euthanized by ethyl ether. The interscapular brown adipose tissue (iBAT) or inguinal white adipose tissue (iWAT) was dissected and used for the following studies.

### Primary brown adipocyte culture

The connective fiber and white adipose tissue were removed from the iBAT, and washed three times with PBS buffer containing 200 U/mL penicillin (Sigma, St. Louis, MO, USA) and 200 U/mL streptomycin (Sigma, St. Louis, MO, USA). Brown preadipocytes were seeded onto 35-mm culture dishes at a density of 8×10^4^ cells/dish, and incubated at 37°C under a humidified atmosphere of 5% CO_2_ and 95% air until confluence. And brown adipocytes were induced to differentiate using DMEM/F12 with 15% FBS, 0.5 mM IBMX; 0.25 mM indomethacin; 2 μg/mL dexamethasone; 1 nM T3, 20 nM insulin for 2 days and subsequently maintained on differentiation media (1 nM T3, 20 nM insulin). Cells were fully differentiated by day 5 post-induction.

### Chemical treatment and vectors infection

For the *in vitro* experiment, brown adipocytes were treated with TM (1 μg/mL) or Tg (1 μM) for 12 h to create ER stress. The recombinant lentiviral interference vector and adenovirus vector of Sirt1 (si-Sirt1, pAd-Sirt1), adenovirus vector of ATF4 (pAd-ATF4), adenovirus vector of mutant ATF4 (pAd-ATF4-mutant), adenovirus vector of Smad3 (pAd-Smad3) and control vectors were purchased from Gene Pharma (Shanghai, China). Brown adipocytes were first infected with pAd-Sirt1 or si-Sirt1 for 24 h or 48 h at the titer of 1×10^9^ IFU/mL to test the effect of the changes of Sirt1 expression on ER stress and apoptosis; followed treated with TM or Tg for 12 h.

### Intracellular calcium and cellular ATP measurements

The intracellular calcium level was measured by using a fluorescent dye Fluo-3 AM (Beyotime Institute of Biotechnology, Nanjing, China) which can across the cell membrane and Fluo-3 formed under canalization by intracellular esterase. Fluo-3 specifically combines Ca^2+^, generating strong fluorescence with an excitation wavelength at 488 nm. After exposed to TM, pAd-Sirt1 or si-Sirt1, brown adipocytes were harvested and washed twice with PBS, and resuspended in 500 μL Fluo-3 AM (3 μM) for 60 min in dark. Then stained cells with DAPI for 5 min. Fluorescence intensity was measured by BD FACScan (BD Biosciences, Franklin Lakes, NJ, USA), and data were analyzed using Cell Quest software (BD Biosciences). For cellular ATP detection, the luciferase-based ATP-assay from Roche (Mannheim, Germany) was used according to the protocol for users.

### Apoptosis measurement

Cell viability was first measured using Cell Counting Kit-8 (CCK-8, Vazyme, Nanjing, China) assay after incubation with TM. Mid-stage and late-stage apoptosis of brown adipocytes were assayed by using Annexin V-FITC/PI apoptosis detection kit (Beyotime Institute of Biotechnology, Nanjing, China) following the User Protocol. At the end of the incubation time, the cells were gently washed with cold PBS and suspended in 500 mL Annexin V binding buffer. After stained twice with 5 mL FITC labeled-Annexin V and 5 mL PI, cells were incubated for 30 min at room temperature in dark. The cells were viewed immediately at room temperature with an inverted fluorescent microscope (Nikon TE2000-U, Japan), and analyzed by BD FACScan (BD Biosciences, Franklin Lakes, NJ, USA). Data were analyzed using Cell Quest software (BD Biosciences).

### Promoter reporter assay and dual luciferase reporter assay

The ATF4 promoter sequence was analyzed using Genomatrix MatInspector. Two fragments containing ATF4–5′ sequences from –890 to –264 relative to the transcription initiation site were sub-cloned into pGL3-basic vector (Takara, Dalian, China). HEK293 cells were cultured in 24-well plates till 80–90% confluence and co-transfected with Renilla plasmid, pGL3-basic or PGL3-ATF4 plasmid (control reporter), and Smad3 overexpression plasmid (pc-Smad3). Cells were harvested 36 h after transfection and detected using the Dual-Luciferase Reporter assay system (Promega, Madison, WI, USA).

### Chromatin Immunoprecipitation (ChIP) assay

Brown adipocytes were prepared for chromatin immunoprecipitation (ChIP) assay using a ChIP assay kit (Abcam, Cambridge, UK) according to the manufacturer's protocol. Primary antibodies of Smad3 (Abcam, Cambridge, UK) or IgG (Abcam, Cambridge, UK) were used. DNA-protein crosslinking complexes were collected, and purified DNA was subjected to qPCR with SYBR green fluorescent dye (Invitrogen, Carlsbad, CA, USA).

### Co-immunoprecipitation (co-IP) assay

HEK293 cells were transfected with plasmids (His-Sirt1 or Flag-Atf4) using X-tremeGEN™ Transfection Reagent (Roche, Switzerland); Cells were then snap-frozen in lipid nitrogen 24 h after transfection. Whole cell lysate was harvested in lysis buffer with protease inhibitor. Cells were then sonicated for 10 sec and the whole cell lysate was pre-clear with Protein A for 2 h and incubated with 2 μg primary antibody overnight at 4°C. Immune complexes were pulled down with Protein A agarose for 2 h at 4°C with shaking. Beads were washed once with lysis buffer and three times with wash buffer, and then eluted by boiling in SDS sample buffer followed by detection of Western blot.

### Total RNA extraction, cDNA synthesis and Real-time PCR

Total RNA was extracted from adipose tissues (iWAT and iBAT) or brown adipocytes using TRIpure Reagent kit (Takara, Dalian, China) according to the manufacturer's instructions. 500 ng of total RNA was reverse transcribed using M-MLV reverse transcriptase kit (Takara, Dalian, China). Primers were synthesized by Invetrogen (Supplementary Table S1, Shanghai, China). Real-time PCR was carried out in StepOnePlus^TM^ System (Applied Biosystems, Carlsbad, CA, USA) with SYBR Green Master Mix (Vazyme, Nanjing, China). The 2^-ΔΔCt^ method was used to quantitate the relative changes in gene expression normalized to β-actin.

### Protein extraction and western blot analysis

Protein from brown adipocytes or brown adipose tissue was extracted using lysing buffer. Protein concentration was determined using BCA Protein Assay kit (Beyotime Institute of Biotechnology, Nanjing, China). Proteins (30 μg) were separated by SDS-PAGE, transferred to PVDF nitrocellulose membrane (Millipore, Boston, MA, USA), blocked with 5% fat-free milk for 2 h at room temperature and then incubated with primary antibodies in 5% milk overnight at 4°C. Sirt1 (ab110304), Sirt2 (ab191383), CHOP (ab179823), GRP78 (ab108615), ATF4 (ab184909), ERDJ4 (ab118282), Bax (ab32503), Apaf-1 (ab32372), UCP1 (ab2384) and PRDM16 (ab106410) antibodies were all purchased from Abcam (Cambridge, UK). Active-Caspase 3 (bs7004), active-Caspase 9 (bs7070), Bcl-2 (bs1511) and GAPDH (ap0063) antibodies were purchased from Bioworld (Nanjing, China). Smad3 (9523S) and IRE1 (3294S) antibodies were purchased from Cell Signaling Technology (CST, Boston, MA, USA). Rabbit HRP-conjugated secondary antibody (Boaoshen, Beijing, China) was added and incubated at room temperature for 2 h. Proteins were visualized using chemiluminescent peroxidase substrate (Millipore, Boston, MA, USA), and then the blots were quantified using ChemiDoc XRS system (Bio-Rad, Hercules, CA, USA).

### Statistics

Statistical analyses were performed using SAS v8.0 (SAS Institute, Cary, NC). Data were analyzed using One way ANOVA procedure. Comparisons among individual means were made by Fisher's least significant difference (LSD). Data were presented as mean ± SEM. *p* < 0.05 was considered to be significant.

## SUPPLEMENTARY MATERIALS FIGURES AND TABLES


