# Supplementary material for: Reducing Smad3/ATF4 was essential for Sirt1 inhibiting ER stress-induced apoptosis in mice brown adipose tissue
Source: Oncotarget. 2016 Dec 20;8(6):9267–79. doi: 10.18632/oncotarget.14035 (PMC5354730; doi:10.18632/oncotarget.14035)
Supplement: Supplementary file 1 [file oncotarget-08-9267-s001.pdf]

Reducing Smad3/ATF4 was essential for Sirt1 inhibiting ER stress-induced apoptosis in mice brown adipose tissue

Supplementary Materials

Supplementary Table S1: Primer sequences for real-time PCR

Gene	RT-PCR primers (5′–3′)
Sirt1	F[a]: TCAGTGTCATGGTTCCTTTGC<br>R[b]: AATCTGCTCCTTTGCCACTCT
Sirt2	F: AATACCCGCTAAGCTGGATGA
	R: TGCATACAGGAGAAGAAACGC
Sirt3	F:CTGGATGGACAGGACAGATAAG
	R:TCTTGCTGGACATAGGATGATC
UCP1	F: ACTGCCACACCTCCAGTCATT
	R:CTTTGCCTCACTCAGGATTGG
PRDM16	F: AGCCCTCGCCCACAACTTGC
	R: TGACCCCCGGCTTCCGTTCA
PGC1-α	F: CATGGATGGCCTATTTGATGAC
	R: CACGGAGAGTTAAAGGAAGAGC
Cidea	F:TGCTCTTCTGTATCGCCCAGT
	R:GCCGTGTTAAGGAATCTGCTG
GRP78	F: ACCTATTCCTGCGTCGGTGT
	R: GCATCGAAGACCGTGTTCTC
Chop	F: GACGCTTCACTACTCTTGACCCTGCG
	R: GGATGTGCGTGTGACCTCTGT
ATF4	F:CCTAGGTCTCTTAGATGACTATCTGGAGG
	R:CCAGGTCATCCATTCGAAACAGAGCATCG
ATF6	F:TACCACCCACAACAAGACCA
	R:TGATGATCCCGGAGATAAGG
Caspase3	F:GGCTTGCCAGAAGATACCGGT
	R:GCATAAATTCTAGCTTGTGCGCGT
Caspase12	F:CTCTAACTGTCGGAGTCTGAGAAACA
	R:TCAGCAGTGGATATCCCTTTG
Bcl-2	F:CTGGTGGACAACATCGCTCTG
	R:GGTCTGCTGACCTCACTTGTG
Bax	F:TGGTTGCCCTTTTCTACTTTG
	R:GAAGTAGGAAAGGAGGCCATC
Smad3	F:ACCATCCCCAGGTCCCTGGATGGCC
	R:AACTCGGCCGGGATCTCTGTGTGGCGT
Smad5	F:CAACACAGCCTTCTGGTTCA
	R:TTGACAACAAACCCAAGCAG
β-actin	F: ACTGCCGCATCCTCTTCCTC
	R: CTCCTGCTTGCTGATCCACATC

F[a]: Forward primer
R[b]: Reverse primer
